# Association between Self-Rated Health and the Ethnic Composition of the Residential Environment of Six Ethnic Groups in Amsterdam

**DOI:** 10.3390/ijerph121114382

**Published:** 2015-11-12

**Authors:** Eleonore M. Veldhuizen, Sako Musterd, Henriëtte Dijkshoorn, Anton E. Kunst

**Affiliations:** 1 Department of Human Geography, Planning & International Development Studies, Faculty of Social and Behavioural Sciences, University of Amsterdam, Nieuwe Achtergracht 166, 1018 WV Amsterdam, The Netherlands; E-Mail: s.musterd@uva.nl; 2 Department of Epidemiology and Health Promotion, Public Health Service Amsterdam, Nieuwe Achtergracht 100, 1018 WT Amsterdam, The Netherlands; E-Mail: hdijkshoorn@ggd.amsterdam.nl; 3 Department of Public Health, Academic Medical Centre, University of Amsterdam, Meibergdreef 9, 1105 AZ Amsterdam, The Netherlands; E-Mail: a.kunst@amc.uva.nl

**Keywords:** neighborhood ethnic composition, own ethnic density, ethnic heterogeneity, self-rated health, spatial scale

## Abstract

*Background*: Studies on the association between health and neighborhood ethnic composition yielded inconsistent results, possibly due to methodological limitations. We assessed these associations at different spatial scales and for different measures of ethnic composition. *Methods*: We obtained health survey data of 4673 respondents of Dutch, Surinamese, Moroccan, Turkish other non-Western and other Western origin. Neighborhood ethnic composition was measured for buffers varying from 50–1000 m. Associations with self-rated health were measured using logistic multilevel regression analysis, with control for socioeconomic position at the individual and area level. *Results*: Overall ethnic heterogeneity was not related to health for any ethnic group. The presence of other Surinamese was associated with poor self-rated health among Surinamese respondents. The presence of Moroccans or Turks was associated with poor health among some groups. The presence of Dutch was associated with better self-rated health among Surinamese and Turks. In most cases, these associations were stronger at lower spatial scales. We found no other associations. *Conclusions*: In Amsterdam, self-rated health was not associated with ethnic heterogeneity in general, but may be related to the presence of specific ethnic groups. Policies regarding social and ethnic mixing should pay special attention to the co-residence of groups with problematic interrelations.

## 1. Introduction

In recent decades, urban societies in Europe have become more ethnically diverse as a result of large-scale immigration. Countries differ both in the composition of their ethnic populations and in the degree of ethnic residential segregation. Segregation rates in the Netherlands, the U.K. and Belgium are higher than in Germany, Austria and France [1]. Within countries, rates of segregation differ between cities and between ethnic groups.

Similar to most other countries, in the Netherlands, the largest cities are the most ethnically diverse. There are substantial differences in the ethnic composition of these cities. In Amsterdam, about half of the population is of non-Dutch origin. The largest ethnic minority groups are Moroccans (9.0 percent of the population), Surinamese (8.5 percent), Turks (5.2 percent) and Antilleans (1.5 percent). In its southeastern district alone, around 100 different nationalities live together [2]. Moroccans and Turks live generally more segregated than other ethnic groups [1].

Much research has been conducted to assess the effects of the ethnic composition of the residential environment on societal outcomes, such as social mobility [3] and integration [4,5]. It has been suggested that diverse neighborhoods would increase inter-ethnic contact, which would influence social mobility and integration positively. However, this suggestion is under pressure by a growing body of evidence contradicting this idea [6,7].

While mixing neighborhoods has been promoted in several European countries to prevent socioeconomic and ethnic segregation, such policies may also be important for their potential impact on population health. If living in ethnically-mixed neighborhoods has an independent effect on health, be it positive or negative, a reconsideration of these policies might be needed.

Many epidemiologic studies have aimed to assess the independent effect of ethnic composition on mental and physical health [8,9,10]. Previous studies have paid particular attention to the effects of ethnic diversity and own ethnic density. Ethnic diversity is defined in most studies as the degree of ethnic heterogeneity within the neighborhood. Bécares *et al.* [11] showed that, for ethnic minorities, living in heterogeneous neighborhoods is associated with improved mental health. A Dutch study in Rotterdam and studies conducted in the U.S. and the U.K. suggest that the mental health of ethnic minorities may be poorest in homogeneous “white” neighborhoods [12,13,14,15]. Gibbons *et al.* [16] came to the same conclusion with respect to self-rated health: in Philadelphia, minorities living in predominantly white communities were significantly more likely to report poor/fair health than those in segregated minority neighborhoods.

Own ethnic density refers to the percentage of co-ethnics in the neighborhood. Research has not yet provided consistent answers on the direction and strength of possible relationship between health and own ethnic density. Some studies, particularly on mental health, suggest own ethnic density to have a positive effect on health [17,18,19]. Other studies found higher own ethnic density to be associated with greater risk of mortality, poor self-rated health and low birth weight [20,21,22], while some studies found no association at all [23,24,25].

The impact of living among co-ethnics may differ by ethnic group, age and gender. Studies focusing on self-rated health found divergent results. Bécares [26] found high own ethnic density to have an inverse association with general health among black Caribbean people, but a positive association among black African people in the U.K. Patel *et al.* [27] reported a positive association among older Mexican Americans in the U.S. Effects may differ by gender, as well. Shaw *et al.* [28] found an inverse association in the U.S. among both black men and women, while among Hispanics, the association was positive among women, but inverse among men.

Previous research suggests that both ethnic diversity and own ethnic density may influence health through several mechanisms, such as effects on: (1) the quality of social support from neighbors; (2) social cohesion within the neighborhood; and (3) experiences of racism or discrimination [29,30,31,32,33]. The “classic” theory suggests better health if a high proportion of the own ethnic group lives in the neighborhood, because of increased social support and less discrimination. For example, Hunt *et al.* [32] showed that people reported less discrimination when living in areas with a high proportion of their own group.

No predominant theory exists on the effects of ethnic diversity. Some argue, in line with social contact theory, that diversity is associated with higher levels of social capital (in terms of social networks, social cohesion and social support) and with greater respect for ethnic differences [34]. Putnam, however, argued that, in line with conflict theory, ethnic diversity results in a pronounced decline in social solidarity and social capital; but he also asked that attention be paid to the “constrict theory”: diversity might reduce both in-group and out-group solidarity and, thus, might impact bridging capital (ties to people unlike you) and bonding capital (ties to people like you) [35]. He found that “in ethnically diverse neighborhoods residents of all races tend to ‘hunker down’. Trust (even of one’s own race) is lower, altruism and community cooperation rarer, friends fewer” ([35], p. 137). Such mechanisms may also have negative consequences on health. In line with Putnam’s argument, Neil and Neil [36] argue that diversity and community sense are inversely related, possibly because people strive for homophily and proximity. According to Putnam [35], in the long run, successful immigrant societies will create new forms of solidarity and more encompassing identities.

In the scientific literature, there is a lack of attention on the presence of specific ethnic groups (other than the own group) in the neighborhood. Most studies on the relationship between health and ethnic composition focus on relations between the majority group and the minority group as a whole. Some studies refer to more refined categories, such as blacks, whites, non-black minorities and mixed [16]. However, especially in cities where many different ethnic groups live together (like Amsterdam), it may be important to distinguish even more ethnic groups and to examine whether the co-residence of specific ethnic groups, in the neighborhood (and relations between these groups) influences social-support mechanisms, social cohesion, experiences of discrimination, and, ultimately, health.

Another potential limitation to previous studies is that the spatial scale used in most studies may be inappropriate [30,37,38]. The generally-used administratively-defined areas (such as counties, census tracts or electoral wards) may in many cases be irrelevant or too large to examine the relationship between ethnic composition and health. Experiences of discrimination and social support might not relate to ethnic composition as measured at the level of entire administrative areas. Frequently, the ethnic composition of residential environments may strongly differ between different parts of administrative areas. As people might be most confronted with people living nearby, ethnic composition measured at a smaller spatial scale may be more appropriate for identifying the effects of the ethnic composition of residential environments on health.

The main aim of this study is to assess associations of ethnic composition and self-rated health among different ethnic groups in Amsterdam. Different dimensions of ethnic composition will be addressed including ethnic heterogeneity, the presence of own ethnic group and the presence of other ethnic groups. We use a spatial approach that accounts for the possibility that observed effects are dependent on the spatial scale that is applied. More specifically, we use bespoke environments (“buffers” created around the respondent) defined at seven different scales, ranging from 50 up to 1000 m in radius, and we measure the ethnic composition of the residential environment according to each buffer size. To our knowledge, this is the first study to examine this relationship using this spatial approach and that therefore could assess potential effects at very small spatial scales.

## 2. Methods

### 2.1. Ethics Statement

The interview survey data were obtained and analyzed in agreement with the rules of the Declaration of Helsinki of 1975. All data are analyzed and stored anonymously. As the Dutch Act on Medical Research Involving Human Subjects (WMO) does not apply to this study, an official approval of this study by the AMC (Academic Medical Centre) Medical Ethics Review Committee (MERC) was not required (MERC Letter W12_069 # 12.17.0084).

### 2.2. Data

The data were obtained from the 2012 Amsterdam Health Monitor conducted by the Amsterdam Public Health Service. The Monitor surveyed 7218 adult inhabitants. Stratified sampling was used to ensure that residents of all districts and age groups within Amsterdam were represented. Data were collected by Internet (46 percent of all respondents), face-to-face interviews (4 percent) and postal questionnaires (50 percent), with an overall response rate of 38 percent. Male respondents between 19 and 34 and non-Western respondents aged 19–34 years showed particularly low response rates. Details of the survey design are described elsewhere [39]. We were allowed to use the data of the respondents that indicated the willingness to participate in future research (4756). After excluding respondents that lived at locations with less than 25 inhabitants within a buffer of 50 m (83), our sample comprised 4673 respondents.

The survey asked respondents about health indicators, such as physical health and mental health, and health determinants, such as smoking, physical activity, housing and neighborhood conditions. Self-rated health was measured by the response to the question “All in all, would you say your health is very good, good, moderate, poor or very poor?” The answers were classified into two categories: very good/good and moderate/poor/very poor. From the same survey, we obtained data on respondent characteristics, including age, sex, ethnicity, marital status, household composition, educational level and a measure of making ends meet (whether the respondent experienced difficulties living on his or her current household income).

To measure the characteristics of each respondent’s residential environment, we used integral demographic and socio-economic registries at the level of six-digit postcodes maintained by the Department of Research and Statistics of the Municipality of Amsterdam. A six-digit postcode area is the smallest geographical unit available. On average, these areas are 50 by 50 m in size and include 10–20 households. For each postcode area, we constructed several variables describing the ethnic composition: ethnic heterogeneity (described by the Herfindahl Index), the percentage of co-ethnics and the proportion of specific ethnic groups. We distinguish between six ethnic categories commonly used in Amsterdam’s data registries: Dutch, Surinamese, Moroccans, Turks, those from other non-Western countries and those from other Western countries.

**Figure 1:**
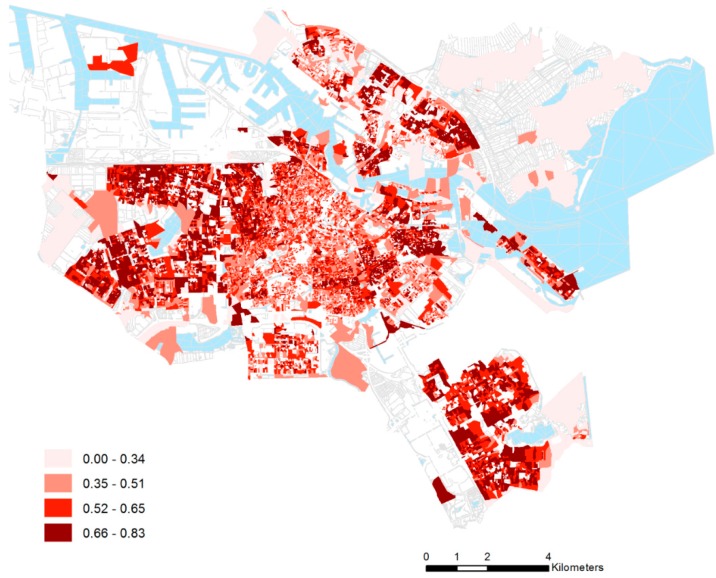
Variations in the degree of ethnic heterogeneity within Amsterdam in 2012 (Herfindahl Index measured for six-digit postcode areas with at least 25 inhabitants).

The Herfindahl Index represents the probability of two randomly-selected individuals from the same neighborhood to differ in ethnic origin. The theoretical range of the index runs from 0–1, with 0 representing an area in which every individual is from the same ethnic group and 1 representing an area in which every individual is from a different ethnic group. To calculate the Herfindahl Index, we sum the squared proportion of each ethnic group and subtract this total from one. Figure 1 shows a map of this index across the 18,111 six-digit postcode areas. The northern, western and (south-) eastern districts are the most ethnically diverse areas within Amsterdam.

To describe the socio-economic environment of each respondent, we constructed two socio-economic variables: The percentage of residents living on a minimum income and the average property value of dwellings.

### 2.3. Construction of Bespoke Environments

Bespoke environments for each respondent were constructed by buffer operations within a geographic information system (GIS). Buffers of seven different sizes, with radiuses ranging from 50–1000 m, were created around the central point of each respondent’s six-digit postcode area as applied previously with similar attention to scale in Veldhuizen *et al.* [40]. Buffers of a 50-, 100-, 150-, 300-, 500-, 750- and 1000-m radius respectively comprise areas of 0.78, 3.14, 7.06, 28.26, 78.51, 176.69 and 314.12 hectares (one hectare is approximately 1.5 soccer fields). The ethnic composition and socioeconomic characteristics of each of these buffers were estimated by aggregating the data of all postcodes belonging to the buffers. More details on the procedure are given in Veldhuizen *et al.* [40].

### 2.4. Statistical Analysis

We assessed the relationship between neighborhood ethnic composition and self-rated health using logistic multilevel regression analysis, with the odds of having moderate/poor/very poor health (“poor health”) measured at the individual level, as the dependent variable. In Model 1, we controlled for age, sex, marital status, household composition, education and a measure of making ends meet at the household level. In Model 2, we additionally controlled for the socio-economic environment at the buffer level, measured with the percentage of households living on minimum income and average property values. The results of these models are expressed in terms of odds ratios, which are derived from the regression coefficients for the ethnic compositional characteristics. The 95 percent confidence intervals are derived from the standard errors of the regression coefficients.

To enable a comparison between the different buffer sizes, we present the odds ratios corresponding to standardized regression coefficients. This procedure is equivalent to transforming the ethnic composition variables into z-scores, for each buffer size separately, before performing a multilevel logistic regression. These standardized odds ratios can be interpreted as the increase in the odds of poor perceived health if ethnic composition were to change with one standard deviation.

## 3. Results

Table 1 describes the characteristics of the study population according to ethnic group. In general, non-Western migrants, particularly Turks and Moroccans, report poor health more often than Western migrants and native Dutch people. Non-Western migrants show higher percentages of single parent families, low education levels and difficulties making ends meet. Western migrants and native Dutch respondents show a higher percentage of people above 65 years old.

**Table 1:** Characteristics of respondents and their socio-economic environment by ethnic group (in percentages).

	Moroccans	Turks	Surinamese	Other Non-Western Migrants	Western Migrants	Dutch
N	148	153	288	288	586	3210
Self-rated health						
Very good	11.6	11.1	13.7	16.1	24.4	20.1
Good	39.7	30.7	45.1	52.3	50.9	55.6
Moderate	30.1	39.9	32.4	22.8	20.1	20.2
Poor	15.1	15.0	7.0	6.0	3.8	3.5
Very poor	3.4	3.3	1.8	2.8	0.7	0.5
Sex						
Male	50.7	34.0	42.0	39.2	36.9	43.2
Age						
19–29	19.6	12.4	16.0	19.8	14.3	15.3
30–39	23.6	30.1	18.1	27.8	22.9	16.2
40–49	19.6	25.5	14.2	21.5	12.6	11.0
50–64	23.6	19.6	30.9	21.9	18.8	20.8
≥65	13.5	12.4	20.8	9.0	31.4	36.7
Marital status						
Married or unmarried couple	68.3	69.3	36.2	50.9	53.2	55.0
Never been married	16.9	12.7	39.1	30.9	28.8	26.5
Divorced	11.3	8.7	20.4	14.7	11.6	9.6
Widow/widower	3.5	9.3	4.3	3.5	6.4	8.9
Household composition						
Two adults with children <18	33.6	37.1	15.7	26.1	16.8	12.2
Single-parent family children <18 years old	8.4	9.9	12.5	10.2	3.3	2.2
Single	12.6	9.9	31.8	22.9	28.8	31.2
Other	45.5	43.0	40.0	40.8	51.1	54.4
Education						
Low	39.5	47.2	11.0	20.1	4.5	6.1
Medium	21.1	18.1	35.3	19.7	17.0	22.8
Medium/High	19.0	22.2	30.4	24.0	23.5	23.9
High	20.4	12.5	23.3	36.2	55.0	47.2
Making ends meet						
Easy	17.7	15.1	14.9	20.4	34.5	37.8
Quite easy	26.5	18.4	32.6	33.7	37.6	39.8
Quite difficult	30.6	35.5	28.8	28.1	20.9	17.3
Difficult	25.2	30.9	23.6	17.9	7.1	5.1
Average property value of houses in postcode of residence	182,863	180,049	184,690	196,414	252,084	243,540
Average percentage of households living on a minimum income in postcode of residence	30.0	27.8	24.4	22.8	14.7	14.0

Table 2 shows, for different ethnic groups, the own ethnic density and ethnic heterogeneity of their residential environments as defined at different spatial scales. On average, Turkish respondents show the lowest share of co-ethnics in their immediate surroundings. The native Dutch respondents have the highest proportion of co-ethnics in their residential neighborhood; on average, Dutch respondents live among over 50 percent co-ethnic Dutch. Own ethnic density generally decreases as buffers increase. Only for the Surinamese group this decrease is modest. The standard deviations for own ethnic density are high, but quickly decrease with increasing buffer size, especially for Turks and Moroccans, indicating that residential environments differ most between respondents when these environments are defined at small spatial scales.

**Table 2:** Mean and variation of own ethnic density and ethnic heterogeneity (Herfindahl) per ethnic group and spatial scale (buffer size).

Buffer	Moroccans		Turks		Surinamese		Other Non-Western		Western		Dutch	
	Mean	SD	Mean	SD	Mean	SD	Mean	SD	Mean	SD	Mean	SD
Own ethnic density												
50	23.13	14.42	16.38	10.50	20.04	15.67	^#^				57.73	18.25
100	21.00	12.45	13.76	7.48	18.73	14.63					55.96	17.13
150	20.18	11.62	13.44	7.15	18.32	13.98					55.04	16.56
300	18.46	10.23	12.08	6.00	17.95	13.56					53.58	15.28
500	17.32	9.20	11.29	5.45	17.85	13.20					52.30	14.15
750	16.18	7.88	10.74	5.18	17.62	13.10					51.52	13.05
1000	15.47	7.32	10.07	4.88	17.39	13.10					51.36	12.27
Ethnic heterogeneity												
50	0.72	0.10	0.72	0.09	0.68	0.11	0.67	0.11	0.59	0.14	0.56	0.16
100	0.73	0.08	0.74	0.08	0.69	0.09	0.68	0.11	0.61	0.13	0.59	0.15
150	0.73	0.09	0.75	0.07	0.69	0.09	0.69	0.11	0.63	0.13	0.60	0.14
300	0.73	0.08	0.75	0.06	0.70	0.08	0.69	0.10	0.64	0.11	0.62	0.12
500	0.73	0.08	0.75	0.06	0.70	0.07	0.69	0.09	0.65	0.10	0.64	0.11
750	0.73	0.07	0.74	0.06	0.70	0.06	0.69	0.08	0.65	0.09	0.65	0.10
1000	0.72	0.07	0.73	0.06	0.70	0.06	0.69	0.07	0.66	0.08	0.65	0.09

^#^ Because the other non-Western migrants and the Western migrants are very heterogeneous groups, it is impossible to measure own ethnic density in an accurate and meaningful way.

Findings on ethnic heterogeneity in the immediate surroundings show that, on average, respondents of Turkish and Moroccan origin live in the most heterogeneous neighborhoods, and Western migrants and Dutch in the least. Generally, larger buffers are more heterogeneous, especially for Western migrants and native Dutch. For Turks and Moroccans, however, heterogeneity slightly decreases beyond buffers of 500 m.

In further descriptions, we assessed the correlations between similar characteristics of the buffers. Because smaller buffers nest into larger buffers, high correlations could be expected. Correlations were highest among buffers of relatively similar sizes. For example, for Moroccans, the percentage of Turks in 50-m buffers was strongly correlated to the percentage of Turks in 100-m buffers (Pearson correlation of 0.854) and more weakly correlated to the percentage of Turks in 1000-m buffers (0.663).

Table 3 shows the associations of own ethnic density and ethnic heterogeneity with self-rated health, for each ethnic group. Only for the Surinamese and Dutch, own ethnic density results are statistically significant. For the Surinamese, we found the higher the percentage of co-ethnics in the neighborhood, the higher the chance they report poor self-rated health. After adjustment for socioeconomic environment (Model 2), the associations remain significant at all distances up to 500 m. Results are consistent for buffers less than 1000 m, and significances are highest for small-sized buffers. However, large buffers of 1000 m come to be important by showing the strongest association. Conversely, for the Dutch, we found that more co-ethnics (Dutch) in the neighborhood decreases the chances of reporting poor self-rated health, but b-coefficients were rather low. Significance decreases with increasing buffer size. After adjustment for socioeconomic environment, the significant results found in Model 1 disappear.

**Table 3:** Association of own ethnic density and ethnic heterogeneity with poor self-rated health ^&^, per ethnic group and spatial scale.

	Own Ethnic Density		Ethnic Heterogeneity	
	Model 1 ^#^	Model 2 ^##^	Model 1	Model 2
Buffer Size	Standardized OR ^$^ (CI)	Standardized OR (CI)	Standardized OR (CI)	Standardized OR (CI)
Moroccans				
50	0.82 (0.46; 1.47)	0.69 (0.37; 1.31)	1.11 (0.58; 2.11)	1.02 (0.50; 2.12)
100	0.85 (0.45; 1.61)	0.80 (0.44; 1.47)	1.15 (0.68; 1.97)	1.08 (0.56; 2.09)
150	1.04 (0.58; 1.86)	0.86 (0.44; 1.69)	1.11 (0.65; 1.88)	1.05 (0.55; 1.96)
300	0.82 (0.47; 1.44)	0.59 (0.30; 1.18)	0.96 (0.56; 1.62)	0.86 (0.47; 1.57)
500	0.75 (0.43; 1.30)	0.54 (0.27; 1.08)	0.94 (0.58; 1.58)	0.78 (0.41; 1.48)
750	0.85 (0.48; 1.48)	0.66 (0.33; 1.29)	0.98 (0.61; 1.64)	0.70 (0.36; 1.36)
1000	0.93 (0.53; 1.62)	0.73 (0.36; 1.47)	1.08 (0.63; 1.81)	0.76 (0.39; 1.50)
Turks				
50	1.41 (0.86; 2.31)	1.31 (0.78; 2.22)	0.97 (0.56; 1.70)	0.86 (0.42; 1.71)
100	1.67 (0.98; 2.84)	1.66 (0.96; 2.84)	1.45 (0.80; 2.61)	1.27 (0.64; 2.53)
150	1.69 (0.99; 2.89)	1.70 (0.99; 2.91)	1.54 (0.90; 2.61)	1.55 (0.85; 2.82)
300	1.78 (1.04; 3.05) *	1.71 (0.98; 2.98)	1.52 (0.90; 2.60)	1.43 (0.77; 2.67)
500	1.53 (0.94; 2.50)	1.37 (0.81; 2.32)	1.34 (0.81; 2.24)	1.37 (0.73; 2.54)
750	1.48 (0.92; 2.41)	1.43 (0.85; 2.40)	1.42 (0.85; 2.37)	1.52 (0.81; 2.84)
1000	1.48 (0.92; 2.41)	1.34 (0.77; 2.32)	1.44 (0.87; 2.36)	1.60 (0.86; 2.97)
Surinamese				
50	1.57 (1.13; 2.20) **	1.57 (1.12; 2.21) **	0.81 (0.55; 1.20)	0.73 (0.49; 1.07)
100	1.58 (1.12; 2.23) **	1.59 (1.12; 2.25) **	0.92 (0.63; 1.33)	0.85 (0.58; 1.25)
150	1.44 (1.03; 2.00) *	1.53 (1.06; 2.20) *	1.04 (0.71; 1.52)	1.02 (0.68; 1.53)
300	1.40 (1.00; 1.96)	1.58 (1.04; 2.40) *	1.22 (0.83; 1.79)	1.40 (0.81; 2.44)
500	1.39 (0.99; 1.96)	1.55 (1.03; 2.33) *	1.33 (0.93; 1.89)	1.30 (0.87; 1.93)
750	1.36 (0.97; 1.92)	1.48 (0.96; 2.28)	1.28 (0.90; 1.83)	1.18 (0.79; 1.77)
1000	1.36 (0.97; 1.91)	2.10 (1.26; 3.48) **	1.40 (0.98; 2.01)	1.36 (0.89; 2.08)
Other non-Western migrants				
50			1.36 (0.98; 1.88)	1.14 (0.79; 1.65)
100			1.51 (1.07; 2.11) *	1.34 (0.91; 1.96)
150			1.47 (1.04; 2.06) *	1.36 (0.93; 1.99)
300			1.41 (1.00; 1.98)	1.24 (0.82; 1.87)
500			1.30 (0.93; 1.82)	1.20 (0.76; 1.88)
750			1.26 (0.90; 1.75)	1.25 (0.79; 1.99)
1000			1.22 (0.87; 1.70)	1.25 (0.79; 1.98)
Western migrants				
50			1.24 (0.94; 1.62)	1.08 (0.80; 1.47)
100			1.10 (0.85; 1.42)	1.04 (0.77; 1.42)
150			1.04 (0.81; 1.33)	1.03 (0.75; 1.42)
300			1.00 (0.78; 1.28)	0.95 (0.68; 1.34)
500			1.00 (0.78; 1.28)	1.07 (0.75; 1.53)
750			1.02 (0.79; 1.30)	1.13 (0.77; 1.65)
1000			1.00 (0.79; 1.28)	1.03 (0.70; 1.51)
Dutch				
50	0.89 (0.81; 0.98) *	0.99 (0.89; 1.10)	1.14 (1.04; 1.26) **	1.04 (0.93; 1.15)
100	0.87 (0.79; 0.95) **	0.97 (0.87; 1.08)	1.17 (1.06; 1.29) **	1.05 (0.94; 1.17)
150	0.86 (0.78; 0.94) **	0.97 (0.86; 1.08)	1.17 (1.06; 1.29) **	1.05 (0.93; 1.17)
300	0.88 (0.81; 0.97) **	1.03 (0.91; 1.16)	1.14 (1.03; 1.25) **	0.98 (0.87; 1.11)
500	0.89 (0.81; 0.98) *	1.03 (0.91; 1.16)	1.12 (1.02; 1.23) *	0.97 (0.85; 1.10)
750	0.90 (0.82; 0.98) *	1.00 (0.88; 1.13)	1.11 (1.01; 1.22) *	0.98 (0.86; 1.12)
1000	0.90 (0.82; 0.99) *	0.99 (0.87; 1.12)	1.11 (1.01; 1.22) *	1.00 (0.87; 1.15)

^&^ Defined as moderate/poor/very poor; ^#^ Model 1 adjusted for age, sex, marital status, household composition, education and difficulties making ends meet; ^##^ Model 2 extended adjustment for socioeconomic environment, measured by the percentage of households living on minimum income and average property value; ^$^ OR represents the standardized odds ratio (*i.e.*, the change in odds with one standard deviation increase in the predictor variable); * significant at the 0.05 level; ** significant at the 0.01 level.

In Model 1, we found positive associations between ethnic heterogeneity and poor self-rated health: the more heterogeneous the environment, the higher the chance to report poor self-rated health. Weak, though significant, results were found only for the Dutch respondents at all buffer distances and for respondents belonging to other non-Western migrants at small distances. After adjustment for socioeconomic environment (Model 2), all significant results disappear.

Table 4 shows associations between the presence of other ethnic groups in the neighborhood and self-rated health among the different ethnic groups after adjustment of socio-economic environment (Model 2). For Turkish respondents, a higher percentage of Moroccans in the neighborhood is associated with a higher chance of reporting poor self-rated health. The statistical significance and strength of this association decreases with increasing buffer size (with the exception of very small buffers of 50 m). For other non-Western migrants, a higher percentage of Moroccans, as well as a higher percentage of Turks is associated with a higher chance of reporting poor self-rated health. This association does not systematically vary according to buffer size. 

**Table 4:** Association presence of other ethnic groups with poor self-rated health, per ethnic group and spatial scale (Model 2).

	Standardized OR ^$^ (CI)					
% Sur	Moroccans	Turks	Surinamese	Other non-Western Migrants	Western Migrants	Dutch
50	1.35 (0.80; 2.29)	0.87 (0.51; 1.49)	1.57 (1.12; 2.21) **	0.97 (0.70; 1.36)	0.90 (0.69; 1.18)	1.03 (0.94; 1.13)
100	1.46 (0.91; 2.34)	0.81 (0.47; 1.40)	1.59 (1.12; 2.25) **	1.04 (0.75; 1.44)	0.91 (0.68; 1.20)	1.03 (0.93; 1.13)
150	1.46 (0.91; 2.36)	0.74 (0.41; 1.35)	1.53 (1.06; 2.20) *	0.98 (0.71; 1.36)	0.95 (0.71; 1.26)	1.01 (0.92; 1.11)
300	1.25 (0.79; 2.00)	0.55 (0.28; 1.07)	1.58 (1.04; 2.40) *	0.88 (0.63; 1.24)	1.02 (0.77; 1.35)	1.02 (0.92; 1.12)
500	1.26 (0.77; 2.06)	0.57 (0.28; 1.15)	1.55 (1.03; 2.33) *	0.90 (0.63; 1.26)	1.11 (0.84; 1.47)	1.00 (0.91; 1.11)
750	1.19 (0.71; 2.02)	0.60 (0.27; 1.32)	1.48 (0.96; 2.28)	0.84 (0.59; 1.20)	1.16 (0.88; 1.54)	0.99 (0.89; 1.09)
1000	1.19 (0.71; 1.97)	0.81 (0.34; 1.91)	2.10 (1.26; 3.48) **	0.74 (0.51; 1.08)	1.18 (0.88; 1.58)	1.00 (0.90; 1.11)
**% Mor**						
50	0.69 (0.37; 1.31)	1.89 (1.07; 3.39) *	0.83 (0.57; 1.19)	1.51 (1.08; 2.10) *	0.86 (0.65; 1.14)	0.98 (0.89; 1.09)
100	0.80 (0.44; 1.47)	4.29 (1.86; 9.92) **	0.73 (0.50; 1.06)	1.55 (1.12; 2.15) **	0.86 (0.62; 1.18)	0.99 (0.89; 1.11)
150	0.86 (0.44; 1.69)	4.58 (1.90; 11.1) **	0.73 (0.50; 1.06)	1.53 (1.09; 2.14) *	0.83 (0.59; 1.15)	0.98 (0.87; 1.09)
300	0.59 (0.30; 1.18)	2.94 (1.48; 5.85) **	0.76 (0.47; 1.23)	1.35 (0.96; 1.90)	0.79 (0.56; 1.11)	0.92 (0.82; 1.03)
500	0.54 (0.27; 1.08)	2.18 (1.22; 3.95) **	0.80 (0.55; 1.17)	1.34 (0.96; 1.87)	0.85 (0.62; 1.17)	0.95 (0.85; 1.06)
750	0.66 (0.33; 1.29)	2.03 (1.17; 3.50) *	0.87 (0.61; 1.25)	1.43 (1.02; 2.00) *	0.87 (0.64; 1.19)	0.99 (0.89; 1.10)
1000	0.73 (0.36; 1.47)	1.76 (0.99; 3.14)	0.77 (0.37; 1.60)	1.50 (1.07; 2.09) *	0.83 (0.62; 1.11)	1.00 (0.90; 1.11)
**% Tur**						
50	0.78 (0.46; 1.33)	1.31 (0.78; 2.22)	0.91 (0.65; 1.26)	1.36 (1.01; 1.83) *	1.00 (0.77; 1.30)	0.97 (0.89; 1.07)
100	0.64 (0.37; 1.11)	1.66 (0.96; 2.84)	0.85 (0.60; 1.20)	1.56 (1.15; 2.11) **	1.06 (0.81; 1.39)	1.04 (0.94; 1.14)
150	0.65 (0.37; 1.13)	1.70 (0.99; 2.91)	0.90 (0.63; 1.28)	1.40 (1.03; 1.89) *	1.07 (0.81; 1.42)	1.04 (0.94; 1.14)
300	0.60 (0.33; 1.11)	1.71 (0.98; 2.98)	0.96 (0.64; 1.43)	1.35 (0.99; 1.84)	0.97 (0.72; 1.30)	0.97 (0.87; 1.07)
500	0.62 (0.32; 1.19)	1.37 (0.81; 2.32)	0.98 (0.68; 1.41)	1.35 (0.99; 1.84)	1.03 (0.79; 1.34)	0.95 (0.85; 1.05)
750	0.73 (0.40; 1.33)	1.43 (0.85; 2.40)	1.01 (0.71; 1.43)	1.49 (1.08; 2.04) *	1.04 (0.79; 1.22)	0.97 (0.88; 1.07)
1000	0.76 (0.42; 1.37)	1.34 (0.77; 2.32)	0.95 (0.62; 1.43)	1.50 (1.09; 2.05) *	0.96 (0.75; 1.25)	1.00 (0.90; 1.10)
**% Dutch**						
50	1.45 (0.70; 2.96)	0.49 (0.24; 1.00)	0.67 (0.43; 1.03)	0.70 (0.46; 1.05)	0.98 (0.72; 1.32)	0.99 (0.89; 1.10)
100	1.20 (0.60; 2.37)	0.43 (0.21; 0.91) *	0.73 (0.47; 1.14)	0.64 (0.42; 0.97) *	1.03 (0.76; 1.40)	0.97 (0.87; 1.08)
150	1.17 (0.59; 2.34)	0.43 (0.21; 0.92) *	0.66 (0.39; 1.13)	0.73 (0.48; 1.10)	1.04 (0.76; 1.42)	0.97 (0.86; 1.08)
300	1.63 (0.83; 3.20)	0.52 (0.27; 1.01)	0.45 (0.26; 0.78) **	0.89 (0.58; 1.39)	1.02 (0.72; 1.43)	1.03 (0.91; 1.16)
500	1.73 (0.85; 3.53)	0.62 (0.33; 1.17)	0.49 (0.30; 0.80) **	0.93 (0.59; 1.47)	0.88 (0.62; 1.25)	1.03 (0.91;1.16)
750	1.57 (0.79; 3.15)	0.57 (0.32; 1.04)	0.47 (0.28; 0.79) **	0.97 (0.63; 1.50)	0.85 (0.60; 1.21)	1.00 (0.88; 1.13)
1000	1.40 (0.70; 2.82)	0.61 (0.33; 1.13)	0.34 (0.20; 0.61) **	1.10 (0.72; 1.67)	0.89 (0.62; 1.27)	0.99 (0.87; 1.12)

^$^ OR represents the standardized odds ratio; * significant at the 0.05 level; ** significant at the 0.01 level; Sur: Surinamese; Mor: Moroccans; Tur: Turks.

For Turkish, Surinamese and other non-Western respondents, the percentage of Dutch in the neighborhood was inversely associated with poor self-rated health (*i.e.*, positively associated with good health). Among Turks and other non-Western migrants, this association was statistically significant only at smaller buffer sizes. In contrast, among the Surinamese, associations were strongest at larger buffer sizes.

## 4. Discussion

Current evidence on the relationship between neighborhood ethnic composition and health is mixed. For Amsterdam, we studied this relationship at different spatial scales and using different measures of ethnic composition. The results suggest that Putnam’s findings regarding the negative impacts of ethnic heterogeneity on social capital and trust of the other and even of members of one’s own group do not universally apply to reporting poor self-rated health. Ethnic heterogeneity and self-rated health were not statistically-significantly associated for any of the six ethnic groups that we distinguished after controlling for the socio-economic composition of the environment. However, the presence of own ethnic group was associated with higher odds of reporting poor health among Surinamese, but not among other groups. With respect to the presence of specific other ethnic groups in the neighborhood, several significant associations were found. For example, a high proportion of Moroccan-origin residents was associated with poor self-rated health of Turkish and other non-Western residents. A higher proportion of Dutch in the neighborhood was associated with a lower chance to report poor health by Turkish and Surinamese residents.

Special attention was paid to the role of spatial scale. In general, stronger relationships were found at lower spatial scales. For Turks, the effect of having Moroccans and Dutch around was most clear in 100- and 150-m buffers. For non-Western migrants, the influence of Turks, Moroccans and Dutch is most pronounced in 100-m buffers. However, there are some exceptions. Among the Surinamese, the influence of the own group is strongest in large, as well as small buffers.

### 4.1. Evaluation of Data and Methodology

One of the strengths of our study is that we could use detailed socio-economic and demographic data from registries at the level of six-digit postcodes, which is the smallest area of observation available, including no more than 10–20 households in urban areas. This level of geographical detail exceeds that of most previous studies on this topic. Moreover, by using bespoke environments instead of administrative areas, we not only address “scale effects”, but also avoid the problem of “boundary effects”, which are associated with the use of administrative areas [41]. While residents living near the boundary of administrative areas are assigned characteristics of the administrative area they reside in, they may be equally affected by characteristics of neighboring administrative areas.

Because larger buffers overlap, the environmental characteristics of respondents are not entirely independent. This may result in an overestimation of the precision and statistical significance of the associations. This problem may not have affected the levels of statistical significance at smaller scales, where buffers rarely overlapped. However, it might have influenced our findings for Surinamese, where we observed a number of associations, especially in larger buffers. As Surinamese are strongly concentrated in the southeastern district of Amsterdam (called “Bijlmer”), these associations may reflect an unidentified “Bijlmer effect”.

We found a higher non-response among lower educated respondents (42.3 percent compared to 20.6 percent among highly-educated respondents) and among non-Western migrants (on average 41.0 percent compared to 24.8 percent among Western respondents). In addition, we could only include those respondents who had indicated willingness to participate in future research. This might have resulted in a selective group of relatively active, engaged and trusting respondents. If these characteristics are associated with the ethnic composition of neighborhoods, selective inclusion of this group could have affected our estimates of the associations between ethnic composition and health.

The administrative classification of the Amsterdam population into “other non-Western migrants” and “Western migrants” (in addition to four defined ethnic groups) reduced the detail with which we could measure ethnic composition. Due to this, we may have missed more specific associations between ethnic composition and self-rated health. This especially applies to the potential effects of the co-residence with specific groups in the “other non-Western migrants” and “Western migrants” categories.

Because of the cross-sectional design of our study, the observed associations may reflect reverse causality or selection effects. Selective migration plays a role if people prefer to move to areas with specific ethnic composition and if this ability depends on health or related characteristics. For example, the inverse associations that we observed may be due to Surinamese preferring to move away from other Surinamese and to Turks preferring to move away from Moroccans. In Amsterdam, however, such selection effects may be modest because spatial mobility in Amsterdam is limited due to lack of appropriate housing for people willing to move as a result of upward social mobility [42]. Moreover, we controlled for a series of socioeconomic measures that may drive residential mobility. However, as there are other health-related factors that could influence neighborhood selection, selection bias and reverse causality could still have influenced the results.

### 4.2. Interpretation and Comparison to Previous Studies

Large ethnic inequalities exist in the overall prevalence of poor health. Good or very good health is reported by 75.8 percent of native Dutch compared to only 41.8, 51.3 and 58.8 percent of Turks, Moroccans and Surinamese (Table 1). These large inequalities contrast with the much smaller ethnic inequalities in mortality, which are driven by migrants’ lower rates of mortality of most cancer types [43,44]. Instead, migrants have higher prevalence rates for most non-fatal diseases, including highly-disabling disease, such as diabetes and arthritis [45]. These inequalities in disease prevalence are not, or only to a minor extent, attributable to potential differences in access or quality of healthcare [46]. More importantly, these inequalities reflect to an important extent the migrants’ lower socioeconomic position and related disadvantages, such as poorer living and working conditions [47]. In addition, disadvantages specifically related to the position of migrant or ethnic minorities, such as the experience of overt or covert discrimination, have been found to be related to poorer physical or mental health [48].

We found no influence of ethnic heterogeneity on self-rated health. This finding is not in line with the theories of Putnam [35] and Neil and Neil [36], which would suggest a higher chance to report poor self-rated health in heterogeneous neighborhoods because of lower social capital in these neighborhood. Our finding better fits into the recently-started debates on whether community connections (including social trust and social capital) are really weaker in ethnically-diverse communities [49].

The results of this study suggest that the other dimensions of ethnic composition are associated with self-rated health in Amsterdam only among particular ethnic groups. This is in line with other studies showing the associations between own ethnic density and self-rated health to differ between different ethnic groups, both in the U.K. [26] and in the U.S. [28].

A previous study of Surinamese, Turkish and Moroccans in the four largest Dutch cities (including Amsterdam) found no association between own ethnic density and psychological distress in any of the three ethnic minority groups examined [25]. Our results regarding Turkish and Moroccans are similar. However, unlike this previous study, we did find associations between own ethnic density and self-rated health among Surinamese. A possible explanation is that the former study used a spatial scale of four-digit postcode areas (on average 2.5 km^2^), whereas we observed these associations for Surinamese at smaller spatial scales.

Our finding that among the Surinamese respondents, the presence of their own group is associated with poor (instead of good) self-rated health is not in line with the classic ethnic density effect, which predicts better health for people living among co-ethnics. This unexpected association may have different causes. First, it is generally known that Surinamese wish to integrate and participate in the Dutch society. From this perspective, living in a district characterized by a high percentage of co-ethnics (the “Bijlmer” district) might represent an undesirable situation, associated with poor self-rated health. Second, residence in areas dominated by Surinamese may imply co-residence among Surinamese subgroups that have poor relations. The Surinamese group includes the Creoles, Hindustani, Javanese and Chinese, with each sub-group having their own organizations, events, meeting places and social networks [50].

The presence of Moroccans in the residential environment was associated with poorer self-rated health for Turks. This association is consistent with explanations derived from identity threat theory. According to this theory, similar groups evaluate each other negatively (compared to other groups) if they threaten each other’s distinctive identities [51]. In the Netherlands, Turks and Moroccans have strong similarities in socio-economic circumstances, migration history, religion and culture [52]. As media attention and general public opinion in The Netherlands is negative about Moroccans, the Turks may perceive their presence as a threat to their position and identity as a minority group. We did not find a reverse association: the co-residence of Turks was not associated with self-rated health of Moroccans. This is consistent with Moroccans not seeing Turks as a threat: Moroccans judge mostly positively about the Turks, whereas Turks judge negatively about Moroccans [53].

### 4.3. Future Research Directions

For future research on relationships between health and the ethnic composition of the residential environment, we recommend a more detailed measurement of ethnic composition. In addition to measuring the general level of ethnic heterogeneity of an area, attention should be given to the presence of other specific ethnic groups, including the respondents’ own group.

Moreover, it is important for future studies to measure the ethnic composition of the residential environment at different spatial scales. Not only should ethnic composition be measured in broad (often administratively defined) areas, such as city districts, but also in the immediate surroundings of respondents’ home address. Different mechanisms may operate at different spatial scales: While integration and discrimination mechanisms may operate at larger spatial scales, social support mechanisms may operate at smaller scales [54].

Finally, extensive adjustment for socioeconomic environment is needed to better understand the role of neighborhood deprivation and to disentangle the effect of deprivation and residential ethnic composition. Our study shows that adequate control for the socioeconomic environment may noticeably alter the observed association between health and ethnic composition.

## 5. Conclusions

In Amsterdam, there is no general association between neighborhood ethnic composition and self-rated health. Instead, such associations are observed only for particular combinations of ethnic groups, especially when these occur in the immediate surroundings of the place of residence. These findings suggest that mixing policies addressing the ethnic composition of areas do not have generalized positive or negative effects on urban health. Instead, our analysis points to localized effects, sometimes positive and sometimes negative, depending on the combination of ethnic groups. Conflict situations in areas where specific groups with problematic inter-relations live together should be addressed for mixing policies to positively contribute to urban health.
